# Gastric Normal Adjacent Mucosa Versus Healthy and Cancer Tissues: Distinctive Transcriptomic Profiles and Biological Features

**DOI:** 10.3390/cancers11091248

**Published:** 2019-08-26

**Authors:** Sabino Russi, Giovanni Calice, Vitalba Ruggieri, Simona Laurino, Francesco La Rocca, Elena Amendola, Cinzia Lapadula, Debora Compare, Gerardo Nardone, Pellegrino Musto, Mario De Felice, Geppino Falco, Pietro Zoppoli

**Affiliations:** 1 Laboratory of Preclinical and Translational Research, IRCCS–Referral Cancer Center of Basilicata (CROB), 85028 Rionero in Vulture (PZ), Italy; 2 Department of Biology, University of Naples Federico II, 80138 Naples, Italy; 3 Pathology Unit, IRCCS, Referral Cancer Center of Basilicata (CROB), 85028 Rionero in Vulture, Italy; 4 Department of Clinical Medicine and Surgery, University of Naples Federico II, 80131 Naples, Italy; 5 Unit of Hematology and Stem Cell Transplantation, IRCCS–Referral Cancer Center of Basilicata (CROB), 85028 Rionero in Vulture, Italy; 6 Istituto per l’Endocrinologia e l’Oncologia Sperimentale “Gaetano Salvatore” (IEOS), Consiglio Nazionale delle Ricerche (CNR), 80131 Naples, Italy; 7 Biogem, Istituto di Biologia e Genetica Molecolare, Via Camporeale, 83031 Ariano Irpino (AV), Italy

**Keywords:** gastric cancer, normal tissue adjacent to the tumor, gene expression profile

## Abstract

Gastric cancer (GC) is a leading cause of cancer-related deaths in the world. Molecular heterogeneity is a major determinant for the clinical outcomes and an exhaustive tumor classification is currently missing. Histologically normal tissue adjacent to the tumor (NAT) is commonly used as a control in cancer studies, nevertheless a recently published paper described the unique characteristics of the NAT in several tumor types. Little is known about the global gene expression profile of gastric NAT (gNAT) which could be an effective tool for a more realistic definition of GC molecular signature. Here, we integrated data of 512 samples from the Genotype-Tissue Expression project (GETx) and The Cancer Genome Atlas (TCGA) to analyze the transcriptome of healthy gastric tissues, gNAT, and GC samples. We validated TCGA-GETx data mining through inHouse gNAT and GC expression dataset. Differential gene expression together with pathway enrichment analyses, indeed, led to different results when using the gNAT or the healthy tissue as control. Based on our analyses, gNAT showed a peculiar gene signature and biological features, like the estrogen receptor pathways activation, suggesting a molecular behavior partially different from both healthy and GC tissues. Therefore, using gNAT as healthy control tissue in the characterization of tumor associated biological processes and pathways could lead to suboptimal results.

## 1. Introduction

Gastric cancer (GC) was the third leading cause of cancer mortality in 2018, responsible for 783,000 deaths (http://www.who.int/news-room/fact-sheets/detail/cancer) and of a poor 5-year survival in case of an advanced stage diagnosis or metastatic disease [1,2]. GCs are mostly adenocarcinomas, subdivided [3] into intestinal and diffuse types according to the Lauren classification and into papillary, tubular, mucinous (colloid) and poorly cohesive carcinomas according to the World Health Organization [4]. In cancer studies, the histological normalcy also implies molecular normalcy. However, this assumption could not be applied to histologically normal adjacent tumor (NAT). It is well known that many molecular differences (versus normal tissues) characterize NAT such as allelic imbalance, telomere length [5], as well as transcriptomic and epigenetic aberrations [6]. Overall, the NAT tissue can be considered an intermediate, morphologically normal but molecularly altered pre-neoplastic state and these changes are evident up to 1 cm from the margins of the tumor [7]. About breast NAT, recent studies reported that the tumor microenvironment is essential for recurrence prediction and surgical strategies setting [8] and that, interestingly, NAT tissue is enriched for stromal [9] and wound response pathways [10]. It has also been highlighted that breast NAT tissue undergoes wound healing-like processes, extracellular matrix remodeling and an epithelial-to-mesenchymal transition (EMT) [11]. Transcriptomic analysis performed on prostate [12], liver [13], and colon [14] cancers also identified unique gene expression profiles for NAT, resulting from a crosstalk between tumor and adjacent tissue, principally mediated by cytokines and other tumor-secreted factors. Thus, comparing tumor and NAT tissues, usually considered as healthy control samples, many potential cancer biomarkers could be missed and/or wrongly pointed out, as it has been recently showed by Aran et al. [7]. Accordingly, based on the above-mentioned studies, we focused on the molecular characteristics of gastric NAT (gNAT), comparing its transcriptomic profile with tumor and non-diseased tissues, hereinafter defined as “healthy normal” samples. To reach our goal, we applied a system biology approach looking for global features like pathways and tissue composition. In particular, we integrated the transcriptomic data from the Genotype-Tissue Expression (GTEx) [15] and The Cancer Genome Atlas (TCGA) [16] projects. Then, we performed a comprehensive analysis of transcriptomic profiles from healthy gastric tissue, gNAT, and gastric tumor, including dimensionality reduction, differential gene expression, gene set enrichment and tissue composition analyses to provide a coherent definition of gNAT molecular phenotype. Remarkably, our analyses highlighted a possible bias depending on suboptimal sampling of normal gastric mucosa. Indeed, a subgroup of normal gastric samples in GTEx was characterized by muscular phenotype according to both gene set enrichment and anatomo-pathological description. Such subgroup shares many similarities with the tumor samples and its inclusion in any further analyses could impinge a clear assessment of the tumor phenotype. Furthermore, we showed that the gNAT tissue is distinct from both healthy and tumor tissues and represents an intermediate state, possibly resulting from NAT-tumor crosstalk.

## 2. Results

### 2.1. Integrative Analysis of TCGA and GTEx RNA-Seq Data

To compare samples’ transcriptomic profiles of TCGA and GTEx projects, we adopted an identical analysis approach. In particular, we obtained raw RNAseq reads of both GTEx and TCGA samples using the same pipeline [17] and compiled a dataset comprising of 162 healthy normal samples, 32 gNAT samples and 380 primary gastric tumor samples (Table 1). On average, cancer patients were older than healthy donors of 18.3 years, while women were 7% more abundant in the healthy group. We assessed differential batch effects and we verified datasets comparability by evaluating the expression and variation of housekeeping genes [18]. We correlated their median expression levels across all samples and we found a high degree of agreement between the two datasets (Pearson R = 0.95, *p*-value << 1 × 10^−6^) (Figure S1).

### 2.2. Evaluation of the Samples Molecular Variability

**Figure 1:**
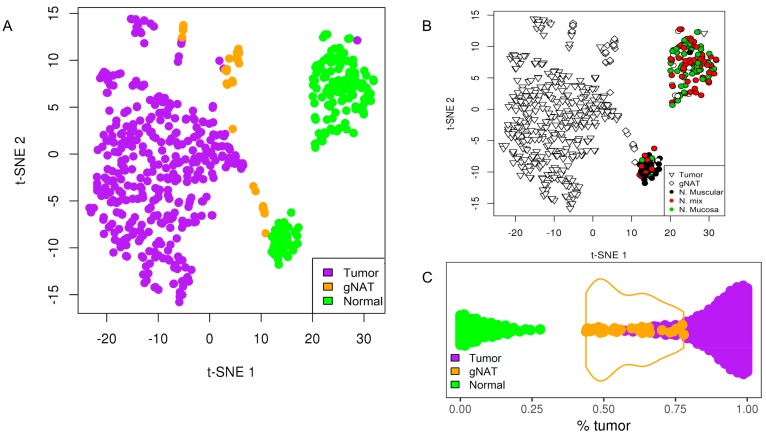
Scatter plot of the samples after dimensionality reduction procedure. (**A**) Gastric cancer (GC) in purple, gNAT in orange and normal tissue in green; (**B**) GC and gNAT with no fill while normal tissue in black red or green according to the anatomopathological classification as muscular, mixed or mucosa. (**C**) Deconvolution highlighting the tumor/normal fraction for each sample. GC in purple, gNAT in orange and normal tissue (only mucosa) in green. In orange violin plot overlay of the gNAT distribution.

In this study, gNAT and healthy tissues data were obtained from TCGA and GTEx datasets, respectively. A major limitation inherent to the integration of multiple independently collected datasets is disparity among sample sets and the different sequencing protocols. By standardizing analysis pipelines according to Q. Wang et al. and removing possible technical distortions such as differences in sample preparation and batch effects through EDAseq and RUVSeq [19,20] packages (Figure S3), data were successfully merged enabling the analyses of RNA-Sequencing data from different sources [21,22]. We investigated the most important sources of variability through a dimensionality reduction process that, reducing the variables (genes) under consideration, enabled us to distinguish three biological groups, with gNAT samples graphically localized between tumor and healthy samples (Figure 1A). Interestingly, the healthy samples were further divided in two clusters, both separated from the tumor and the gNAT groups, one resulting to be more similar to the tumor along the first component. According to the anatomopathological classification of the samples, it resulted to be composed by muscular tissue more than by gastric mucosa (Figure 1B), thus we named the two normal clusters as “Normal Muscular” and “Normal Mucosa”.

Furthermore, we comparatively evaluated the two clusters through differential expression and gene enrichment analyses. Among the differentially expressed genes (DEGs), we found 2711 genes up regulated in mucosa cluster enriching gastric acid secretion and 2568 up regulated genes in muscular cluster enriching vascular smooth muscle contraction (Figures S4 and Figure S5). Moreover, the heatmap of the top 900 DEGs (adjusted *p*-value << 1 × 10^−6^ and abs (log_2_FC) > 3) was reported in Figure S6. However, since the muscular samples are not representative of the normal gastric tissue, we considered not appropriate to include them in further analyses. As above mentioned, gNAT samples appeared distinguished from the other tissues’ samples. As shown in Figure 1C, by using a deconvolution pipeline [23] able to calculate the “normal:tumor” fraction for all samples, we found that gNAT samples were positioned between tumor and healthy tissue samples. Strikingly, gNAT group was similar to the tail of the tumor sample distribution, possibly suggesting a microscopic contamination of gNAT samples with tumor and vice versa. However, even considering such overlap, gNAT appeared to be a distinct tissue type. To validate our findings, we searched public data repositories for independent studies that collected samples from all three tissue types included in our analyses. Our search yielded a microarray cohort (E_MTAB_1338) with sufficient sample sizes of GC, gNAT, and healthy tissues. The same methodology described above applied on this dataset resulted in a consistent distribution of samples (Figure S7). Consistently with the TCGA-GTEx samples pattern (Figure 1A), even in this small dataset, we found two healthy tissue clusters and one of the subgroups characterized by the activation of the gastric acid secretion (adjusted *p*-value < 0.01), as pointed in Figure S8.

### 2.3. Evaluation of the Adequacy of the gNAT as Control in Cancer Research

**Figure 2:**
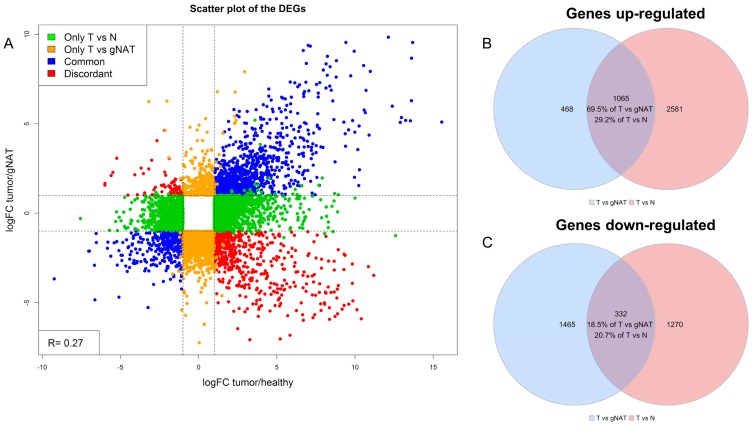
Tumor (T) vs. gNAT and T vs. normal differentially expressed genes. (**A**) Scatter plot of the log_2_FC. DEGs in green exclusively in T vs. N, in orange exclusively in T vs. gNAT, in blue in common while in red discordant. (**B**) Overlap of the up regulated genes. (**C**) Overlap of the down-regulated genes.

To assess the impact of different/sub-optimal control samples on differential expression analyses results, we performed further evaluations. Firstly, we compared results obtained by considering gNAT and healthy normal samples as controls for tumor samples. Comparison between tumor and gNAT resulted into 3330 DEGs while 5248 DEGs were obtained by comparing tumor with normal samples. Interestingly, the overall Pearson correlation between fold-changes was poor (R = 0.27), suggesting that gNAT and healthy normal samples have significantly different molecular signatures. As depicted in Figure 2A, among the DEGs resulted from the comparative analysis, 1397 genes showed the same behavior, 634 DEGs were discordant, while 3217 and 1299 were significant only in one of the two comparisons. Overlap between up and down regulated genes deriving from the comparisons was depicted in the Venn diagrams in Figure 2B,C.

**Figure 3:**
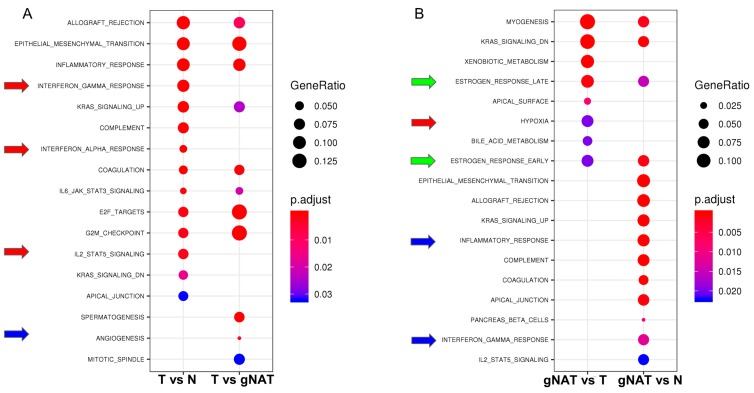
Gene set analysis (GSA) of the hallmark gene sets using the differentially expressed genes (DEGs) between tumor or gNAT vs. each of the other two tissues, respectively. Adjusted *p*-value in red-blue color scale. Gene ratio in dot size scale. Red, blue and green arrows highlight interesting exclusive and common gene sets, respectively. (**A**) Tumor centered analysis, (**B**) gNAT centered analysis.

To investigate whether using different control samples could have an impact on the biological features associated to GC, we performed a hypergeometric test using the 50 hallmark and the 5917 Gene Ontology (GO) gene sets from mSigDB [24]. Altogether, 8 out of 17 significant (adjusted *p*-value < 0.05) hallmarks were in common, 6 were specific of the comparison of tumor samples with normal and 3 of tumor vs. gNAT (Figure S9A). In particular, using the gNAT as control masked some hallmarks such as interferon response or the IL2/STAT5 signaling also pinpointing misleading results as angiogenesis (Figure 3A). When we compared the GO enrichments, only 220 out of 1214 (adjusted *p*-value < 0.05) were in common, 863 were specific of the comparison with normal and 131 of the comparison with gNAT (Figure S9B). In particular, the gNAT masked some GO as innate and adaptive immune responses (Figure S10). Overall, we concluded that considering gNAT as a control for GC samples did not allow detection of the majority of DEGs in tumors neither accurately identified all the biological features of the GC.

### 2.4. Molecular and Biological Characterization of the gNAT

**Figure 4:**
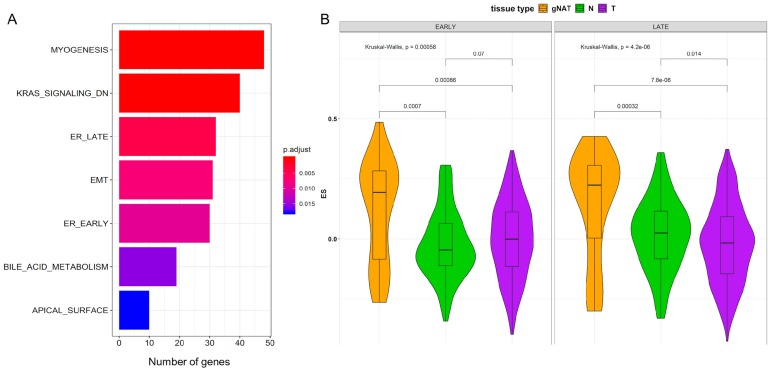
(**A**) GSA of the hallmark gene sets using the genes up regulated in gNAT vs. both the other 2 tissues. Adjusted *p*-value in red-blue color scale, (**B**) Violin plot of the hallmark early and late estrogen receptor (ER) pathways activity in gNAT, normal and tumor, respectively in orange, green and purple. ES obtained by gene set enrichment analysis (GSEA).

To further explore the divergence between gNAT and tumor samples, we performed a differential expression analysis. By comparing gNAT with tumor samples, we identified 1797 upregulated and 1533 downregulated DEGs (Tables S1 and S2). To gain further insight into the global patterns that differentiate the gNAT from tumor specimens, we performed a gene set analysis (GSA) using the hallmark and the GO gene sets [24]. GSA on the hallmark and the GO gene sets showed 16% of the all hallmarks and 6% of the all GO, respectively, as significantly different between gNAT and tumor (adjusted *p*-value < 0.05). In particular, hypoxia together with early and late estrogen receptor (ER) pathways hallmarks specifically resulted from this comparison (Figure 3B). Remarkably, many categories have been identified through GO enrichment (Figure S10B). We also assessed DEGs between gNAT and healthy tissue, finding 5415 DEGs (3711 upregulated and 1704 down regulated) (Tables S1 and S2). Altogether, 14 hallmarks and 911 GO were significantly perturbed (adjusted *p*-value < 0.05). Hallmark enrichment analysis highlighted, among the others, early and late ER pathways as well as inflammatory and interferon gamma responses (Figure 3B). Remarkably, among the GO interesting categories, inflammatory and innate immune responses appeared to be exclusive of gNAT vs. healthy tissue comparison while extracellular matrix emerged from both comparisons (Figure S10B). Finally, 1323 genes have been found significantly up regulated in gNATs when compared with both tumor and normal gastric tissue samples. GSA performed on the significantly up regulated genes resulted in 7 activated hallmark categories (Figure 4A), possibly highlighting the molecular features of the gNAT.

Interestingly, early and late estrogen responses together with extracellular matrix seemed to exclusively characterize gNAT (Figure 3B). Inflammatory response, although characteristic of gNAT in its comparison with normal tissue, has not been found enriched in the comparison with tumor. Consistently, a lower expression of estrogen receptors in GC compared to gNAT was reported in literature, confirming our findings [25,26]. Moreover, gene set enrichment analysis (GSEA) on the hallmark categories also confirmed early and late responses to the estrogen as active biological processes (Table S3). In order to shed light on those hallmarks, a single sample gene set enrichment analysis (ssGSEA) was performed on TCGA dataset, as shown in Figure 4B. To further characterize the role of ER pathways in gNATs and GCs we performed univariate analysis of the most interesting clinical variables (grade, TNM, anatomic positions, etc.) (Table S4). Interestingly, we found that both ER pathways were significantly (*p*-value < 0.001) associated with tumor histological grade (Figure S11). In particular, lower enrichment scores (ES) were observed in G3 grade while gNATs showed higher, although not significant (probably due to the few numbers of gNATs), ESs levels. Very interestingly, the therapy success (Table S4) and the anatomic localization of the samples (Figure S12A) significantly correlated with the ES of both ER pathways in gNATs. One criticism exploring the ER response can be related to the samples gender but there is no association between ER activity and gender (Figure S12B). In Figure S13 is shown the association of tissue type and grade with late ER pathway activity according to gender (A) or Japanese Gastric Cancer Association (JCGA) anatomical site (B), respectively. Interestingly there was a strong and significant difference in the distribution of the activity of the ER late pathway between tumor and gNAT in patients with G3 grade proximal/middle localized tumor.

### 2.5. Hypothesis Validation Through inHouse GC RNAseq Dataset Generation

**Figure 5:**
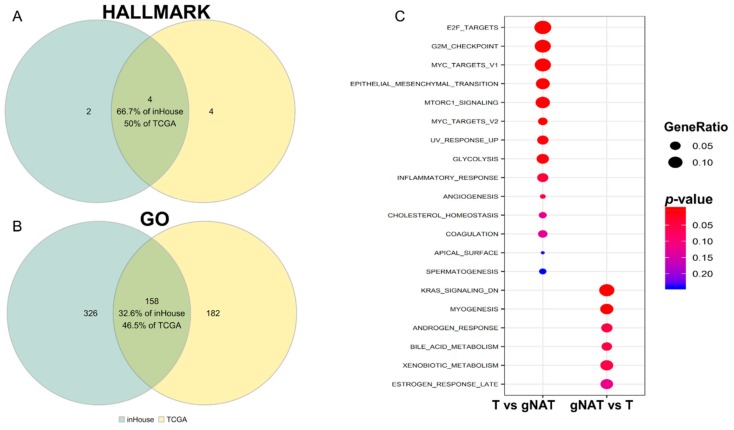
Distribution and description of enriched hallmark and GO gene sets in gNAT vs. Tumor between inHouse and TCGA dataset. (**A**) depicts the hallmark results, (**B**) depicts the GO results, (**C**) depicts GSA results. The *p*-value in red-blue color scale. Gene ratio in dot size scale.

In order to validate our approach, we compared, after performing a quality control (Figure S14), the global gene expression profiles of 9 GC and 9 gNAT samples through RNAseq analyses (inHouse dataset). There were 2563 DEGs (abs (log_2_FC) > 1 and adjusted *p*-value < 0.05), 1625 were found up regulated in tumor vs. gNAT comparison and 938 up regulated in gNAT vs. tumor. Figure 5C shows the hallmark and GO significantly activated categories in both inHouse tumor vs. gNAT and gNAT vs. tumor comparisons. Considering the reduced size of the inHouse samples, compared to the TCGA dataset, we set the threshold at *p*-value < 0.05. As reported in  Table S5 and Figure 5, good overlaps between the tumor vs. gNAT upregulated genes in TCGA and inHouse datasets as well as between (A) the hallmark categories (4 out of 6 and 8) and (B) the GO categories (158 out of 484 and 340) were observed. 

In order to confirm the reliability of the inHouse RNAseq, we processed our 18 samples by digital PCR, analyzing the expression of 6 genes: 1 housekeeping, 1 non-expressed gene and 4 gNAT highly expressed genes, the latter belonging to the hallmark categories enriched in both TCGA and inHouse datasets (myogenesis, late ER and KRAS signaling down). The selection of a good internal standard genes is well documented problem and the suitable genes vary according with tissue specificity and treatments [27]. Here, we selected DEED as housekeeping being its expression among the less variable in TCGA-GTEx dataset. Correlation between digital PCR and RNAseq expression levels was remarkably high (R > 0.97) and the “non-expressed” gene was not detected by digital PCR, as well (Table S6).

## 3. Discussion

Our study provides insight into differences among gNAT, GC, and healthy tissue samples as concerns the gene expression profiles. Importantly, we found that gNAT samples are characterized by a peculiar biological behavior different from both healthy and GC tissues. Hence, considering gNAT specimens as control of tumor samples in GC, molecular characterization studies could be affected, by some extent, by false positive and negative as also recently reported in literature for other types of cancer [7]. Using a dimensional reduction procedure, we realized that some of healthy tissue samples were more similar to the muscle tissue than to the gastric mucosa. This finding could be explained by the quality of samples and the collection procedures (few amounts of specimens and/or quick degradation of gastric mucosa). In order to ensure the suitability of normal samples for an appropriate comparison, we decided to discard the muscular-like ones from further analyses. Subsequently, we highlighted the existence of qualitative and quantitative differences in GC DEGs detected by using gNAT or healthy tissue as control. Correlation between the fold change profiles was low and most genes were discordant or at least non-concordant. Interestingly, some gene sets emerged from comparisons of tumor vs. healthy tissue samples. In particular, interferon response and IL2/STAT5 signaling pathways (Figure 3A) as well as innate and adaptive immune responses (Figure S10A) were missed when comparing tumor with the gNAT specimens. Similarly, angiogenesis (Figure 3A) was detected only in tumor vs. gNAT comparison. Accordingly, we assumed that gNAT is not an appropriate control in gene expression studies aimed to characterize GC molecular signatures and biological features. To better pinpoint gNAT specific characteristics, we examined, firstly separately then together, the gene expression differences among gNAT, healthy gastric mucosa and tumor samples. Comparing gNAT vs. healthy tissue samples (Figure 3B), the enrichment analysis of the hallmark categories highlighted activation of early and late estrogen as well as inflammatory and interferon gamma responses. Remarkably, following GO enrichment (Figure S10B), extracellular matrix, inflammatory and innate immune responses were highlighted. On the other side, considering the differences between gNAT and tumor tissue specimens (Figure 3B), the enrichment analysis identified the activation of hallmark categories like hypoxia together with early and late ER pathways. Interestingly, GO enrichment (Figure S10B) highlighted results similar to those obtained by the comparison of gNAT vs. normal. Moreover, we showed that many gNAT gene profiles were distinct from both healthy and tumor tissues, among which we identified a set of genes specifically overexpressed in gNAT, demonstrating their association with hallmark and GO categories. Therefore, GC gene expression studies not including healthy tissues as control could lead to misidentify gNAT specific genes as selectively under-expressed in the tumor, despite their normal expression levels. Among the results of our analyses, detection of early and late ER pathways activation appeared a quite intriguing finding. In particular, early and late estrogen response gene sets seem to be significantly enriched in the gNAT samples from proximal/middle sites when compared to those from distant sites. No differences on anatomical origin were instead detected among tumor samples. Since in GTEx dataset stomach anatomical sites annotations are missing, it would be useful to consider such information in future investigations. The role of ERs in gastric cancer, possible mechanisms underlying it and clinical relevance of deregulated ERs in GC patients have been widely investigated. Interestingly, ER-α and ER-β have been found to be down regulated in tumor tissues when compared to adjacent mucosa samples [26]. Intriguingly, our results suggested an activation of the ERs pathways in proximal/middle adjacent mucosa compared with tumor tissue samples showing, instead, ER normal expression levels. This finding could be explained by the ability of tumor mass to act as an endocrine organ, controlling metabolism and homeostasis of both neighboring and distant tissues [28]. Noteworthy, several studies demonstrated the presence of high expression and activity levels of estrogen production enzymes that could drive the secretion of biologically active estrogens in GC [29,30]. As for any computationally based study, independent confirmation is necessary to draw conclusions. For this reason, we analyzed 9 gastric tumor and 9 gNAT samples producing a RNAseq dataset (inHouse dataset) on which we performed differential expression analysis. The DEGs, hallmark and GO categories obtained from InHouse analyses were consistent with those resulting from TCGA.

## 4. Materials and Methods

### 4.1. Data Collection and Processing

The Cancer Genome Atlas (TCGA) (https://www.cancer.gov/tcga) and The Genotype-Tissue Expression (GTEx) Project (https://www.gtexportal.org/home/) repositories collected high throughput and clinical data of many tumors and normal tissues from many organs. Although unifying cancer and normal RNA sequencing data from different sources represent a bioinformatics challenge, TCGA and GTEx RNAseq data were successfully merged by Q. Wang et al. enabling cross-study analysis of RNA-Sequencing data [21,22]. From Memorial Sloan Kettering repository (https://github.com/mskcc) and from UCSC Xena browser (https://xenabrowser.net/datapages/) we retrieved dataset (TCGA STAD and GTEx) and clinical data, respectively. In particular, after crossing RNAseq dataset and clinical data, we obtained the data of 380 gastric cancer RNAseq, 32 adjacent normal tissue and 162 normal samples. It is important to pinpoint that all the samples we call “normal” were collected by GTEx from non-diseased tissue sites. Gene annotation retrieved from TCGA using TCGAbiolinks [31] package. All the information about TCGA samples’ clinical data, pathology reports and tissue are easily retrievable by the cBioPortal (https://www.cbioportal.org/) website while the “GTEx Tissue Harvesting Work Instruction” provides all the available information about the GTEx tissues.

To reduce the GC-content bias and standardize the distribution of the counts in each sample we applied EDAseq [32] normalization pipeline adjusting within-lane gene specific effects (GC-content) and between-lane distributional differences (global-scaling using the upper-quartile). Batch effects and differences in sample preparation can have substantial ramifications on the outcomes. Thus, we used stringent removal of unwanted variation, in particular we employed the RUVg method from the RUVSeq package [19], which performs factor analysis on residuals using a negative gene set that has constant covariates. The negative set we used was a list of housekeeping genes [18], and the factors of unwanted variation were added in the design matrix for the regression-like model used by edgeR [33] package to perform differential expression analysis. From ArrayExpress repository [34] we retrieved the 108 samples E-MTAB-1338 [35] dataset containing 71 GC, 21 gNAT, and 16 normal mucosa tissue. Such data were normalized using quantile normalization from beadarray [36] package. Ten GC and 10 gNAT samples obtained from IRCCS-CROB bio-bank (Ethical committee approval N: 20180042426) populated the inHouse dataset (submitted to ArrayExpress repository with ID: E-MTAB-8135).

### 4.2. inHouse Gene Expression Profiles

For library preparation, a barcoded cDNA library first generated with SuperScript® VILO™ cDNA Synthesis kit (Life Technologies Corporation, Carlsbad, CA, USA) from 10 ng of total RNA. Then cDNA amplified using Ion AmpliSeq™ Transcriptome Human Gene Expression Kit (Life Technologies Corporation ) to accurately maintain expression levels of all targeted genes. The average size of each amplicon is ~150 bp. Amplified cDNA Libraries evaluated for quality and quantified using Agilent Bioanalyzer High sensitivity chip. Libraries were then diluted to 100pM and pooled equally, with eight individual samples per pool. Pooled libraries amplified and enriched by using IonChef Instrument (Life Technologies Corporation) according to manufacturer instructions. Templated libraries sequenced on S5™ sequencing system using Ion 540 Kit-Chef kit and chip, obtaining the normalized counts as result of the Target Amplicon-seq pipeline. Moreover, we performed a multidimensional scaling plot of the samples and according to Figure S14 we discarded the “2” (T and S) paired samples as the 2S sample resulted different from the other gNATs.

### 4.3. Dimensionality Reduction

Dimensionality reduction performed using the Rtsne [37] package on the log2 CPM values (RNA-seq), or log2 expression values (microarray). The deconvolution procedure was performed using the DeconRNASeq [23] package and the result of this procedure is a proportion of the “tumor contribution” to the sample.

### 4.4. Differential Expression Analysis and Venn 

Differential expression analysis performed using edgeR. For both RNAseq data, only genes with at least 10 reads in half of the smallest group were included for the analysis, also to handle the pronounced differences in library sizes between TCGA and GTEx (Figure S2). A gene was considered as differentially expressed (DEG) if (1) corrected (FDR) *p*-value < 0.05 and (2) > 2-fold (log_2_FC > 1) expression change. All the comparisons depicted in the Venn diagrams refer to the 9954 genes (universe) overlapping between inHouse and TCGA dataset.

### 4.5. Gene-Set Enrichment Analyses

MSigDB [24] Hallmark, GO and KEGG [38] gene sets overrepresentation for each different DEGs lists obtained applying ClusterProfiler [39] and pathview [40] packages. We considered statistically significant the gene sets resulting from the analysis of TCGA-GETx data with a FDR adjusted *p*-value < 0.05 while, to compensate the relative lower number of samples in the inHouse dataset, we accepted *p*-value < 0.05 as threshold. ssGSEA implemented in the GSVA [41] package was used to score samples according to the normalized counts of the genes on MSigDB Hallmark and GO gene sets. The enrichment score (ES) reflects the degree to which a gene set is overrepresented at the top or bottom of a ranked list of genes. Higher the ES, more activated the pathway/biological process. 

Statistical analysis performed using the computing environment R [42].

### 4.6. Digital PCR

From 1000 ng of total RNA reverse transcribed with SuperScript IV VILO Master Mix (Invitrogen), according to manufacturer’s protocol, about 10 ng of cDNA were used. Droplet Digital PCR (ddPCR) was performed using the QX200™ Droplet Digital™ PCR system (Bio-Rad Laboratories, Hercules, CA, USA) including the droplet generator and the reader. Reactions were prepared in 20 µL volumes with QX200™ ddPCR™ EvaGreen Supermix (Bio-Rad Laboratories) and the following primer sets, retrieved from hg19 AmpliSeq Transcriptome 21K_v1 DataSheet, were used: NCAM1 (Fw-TGTGGACATCACCTGCTACTTC, Rev-ATGGGCTCCTTGGACTCATC); TFF2 (Fw-AGTGCTGCTTCTCCAACTTCAT, Rev-TGATAAGGCGAAGTTTCTTCTTTGGT); LTF (Fw-CCTGTCAGCTGCATAAAGAGAGA, Rev-GTAGACTTCCGCCGCTACA); TFF1 (Fw-GCCCTCCCAGTGTGCAAATAA, Rev-GCCCTCCCAGTGTGCAAATAA); CYP1A1 (Fw-CATCCGGGACATCACAGACA, Rev-GAGATAGCAGTTGTGACTGTGTCAA). We used DEDD (Fw-TCAGATGTGTAGCAAGCGGC, Rev-CAGTATTCAGCCCGAACCCG) as housekeeping gene being one of the most stable in TCGA dataset (data not shown). After droplets generation in DG8^TM^ Cartridges (Bio-Rad Laboratories), ddPCR™ 96-Well PCR Plates containing reactions in duplex were loaded onto Verity 96-well Thermal Cycler (Applied Biosystems, Foster City, CA, USA) and cycled as follows: 5 min at 95 °C, 40X (30 sec at 95 °C, 60 s at 60 °C), 5 min at 4 °C, 5 min at 90 °C, and held at 4 °C. QuantaSoft Software v1.7 (Bio-Rad Laboratories) was used to analyze the output of QX200^TM^ Droplet Reader (Bio-Rad Laboratories). 

## 5. Conclusions

Our results suggest that using gNAT tissue as control in gene expression analysis could mislead the identification of tumor important pathways. Although gNAT as the control tissue in gene expression analysis allows the identification of the major part of tumor biomarkers and pathways, our results suggest that healthy tissue as control improves the molecular and biological characterization of GC. Interestingly, gNAT tissue shows gene profiles and some biological pathways distinct from both healthy tissue and tumor, although the underlying mechanisms remain to be validated. As supported by recent reports, our findings warrant further investigation on the complex interplay between gNAT and GC tissue. Understanding molecular mechanisms orchestrating this crosstalk could indeed pave the way for identification of novel tumor biomarkers and druggable targets for treatment of GC.

## Figures and Tables

**Table 1:** Distribution of samples and demographic characteristics of patients and healthy donors included in the study. GTEx: Genotype-Tissue Expression project; TCGA: The Cancer Genome Atlas; gNAT: gastric normal tissue adjacent to the tumor.

Tissue Type	Number of Samples	Sex (% of Female)	Age (Mean ± SD)
GTEx healthy	162	42.6	47.4 ± 12.4
TCGA gNAT	32	34.4	66.4 ± 9.1
TCGA tumor	380	35	65.7 ± 10.6

## Supplementary Materials

The following are available online at https://www.mdpi.com/2072-6694/11/9/1248/s1, Figure S1: Scatter plot of the correlation of the median expression levels of housekeeping genes in normal and gNAT samples. Greater the standard deviation (SD), greater the radius of the points, Figure S2: Boxplot of the library reads in TCGA GC and gNAT and in GTEx normal samples; GC in purple, gNAT in orange and normal tissue in green, Figure S3: Boxplot of the relative log expression (RLE) pre and post (EDAseq) normalization. A shows the relative log expression (RLE) without normalization. There are differences in RLE between TCGA (GC (purple) and gNAT (orange)) samples and GTEx healthy samples (green) while in B, after normalization, no apparent differences are observed, Figure S4: KEGG enrichment of the vascular smooth muscle contraction pathway (hsa04270) using genes up regulated in normal muscular vs. normal mucosa, Figure S5: KEGG enrichment of the gastric acid secretion pathway (hsa04971) using genes up-regulated in normal mucosa vs. normal muscular, Figure S6: Heatmap of the top 900 DEGs between normal mucosa vs. normal muscular samples. log2FCs in red-green color scale. In legend, above the HM, normal mucosa in cyan while normal muscular in red, Figure S7: Scatter plot of the E_MTAB_1338 samples after dimensionality reduction procedure. (A) GC in purple, gNAT in orange and normal tissue in green, Figure S8: KEGG pathways enrichment in E_MTAB_1338 database using DEGs between the right-low cluster and the middle-high cluster of normal samples, respectively. A Gastric acid secretion pathway (hsa04971). B Vascular smooth muscle contraction pathway (hsa04270). Only the Gastric acid secretion pathway is significantly enriched, Figure S9: Overlap of the enriched hallmark and GO gene sets; A and B depict the results of the overlap between gNAT vs. normal and gNAT vs. GC; C and D depict the results of the overlap between GC vs. gNAT vs. GC vs. normal, Figure S10: gene set analysis (GSA) of the GO categories using the DEGs between tumor or gNAT vs. each of the other 2 tissues, respectively. Adjusted p-value in red-blue color scale. Gene ratio in dot size scale. Red, blue and green arrows highlight interesting exclusive and common gene sets, respectively. (A) Tumor centered analysis, (B) gNAT centered analysis, Figure S11: Association of the histological grade with hallmark early and late ER pathways activity in gNAT and GC, Figure S12: Association of JGCA anatomical part (A) and gender (B) with hallmark early and late ER pathways activity in gNAT and GC, Figure S13: Association of therapy tissue type and grade with hallmark late ER pathway activity according to gender (A) or JCGA anatomical site (B), respectively, Figure S14: multidimensional scaling plot of the RNA samples in which distances correspond to leading log-fold-changes between each pair of RNA samples. GC in purple, gNAT in orange, labels represents the name of the samples, Table S1: Summary of the differentially expressed genes among adjacent, normal and tumor samples., Data S1: TCGA DEGs tumor vs. adjacent vs. normal, Data S2: Enriched hallmark in TCGA gNAT, Data S3: univariate analysis of the most interesting clinical variables (grade, TNM, anatomic positions, etc.) on the TCGA data, Data S4: inHouse DEGs in GC vs. gNAT, Data S5: digital PCR validations.
