# Supplementary material for: Gastric Normal Adjacent Mucosa Versus Healthy and Cancer Tissues: Distinctive Transcriptomic Profiles and Biological Features
Source: Cancers (Basel). 2019 Aug 26;11(9):1248. doi: 10.3390/cancers11091248 (PMC6769942; doi:10.3390/cancers11091248)
Supplement: Supplementary file 1 [file cancers-11-01248-s001.zip › supplementary.docx]

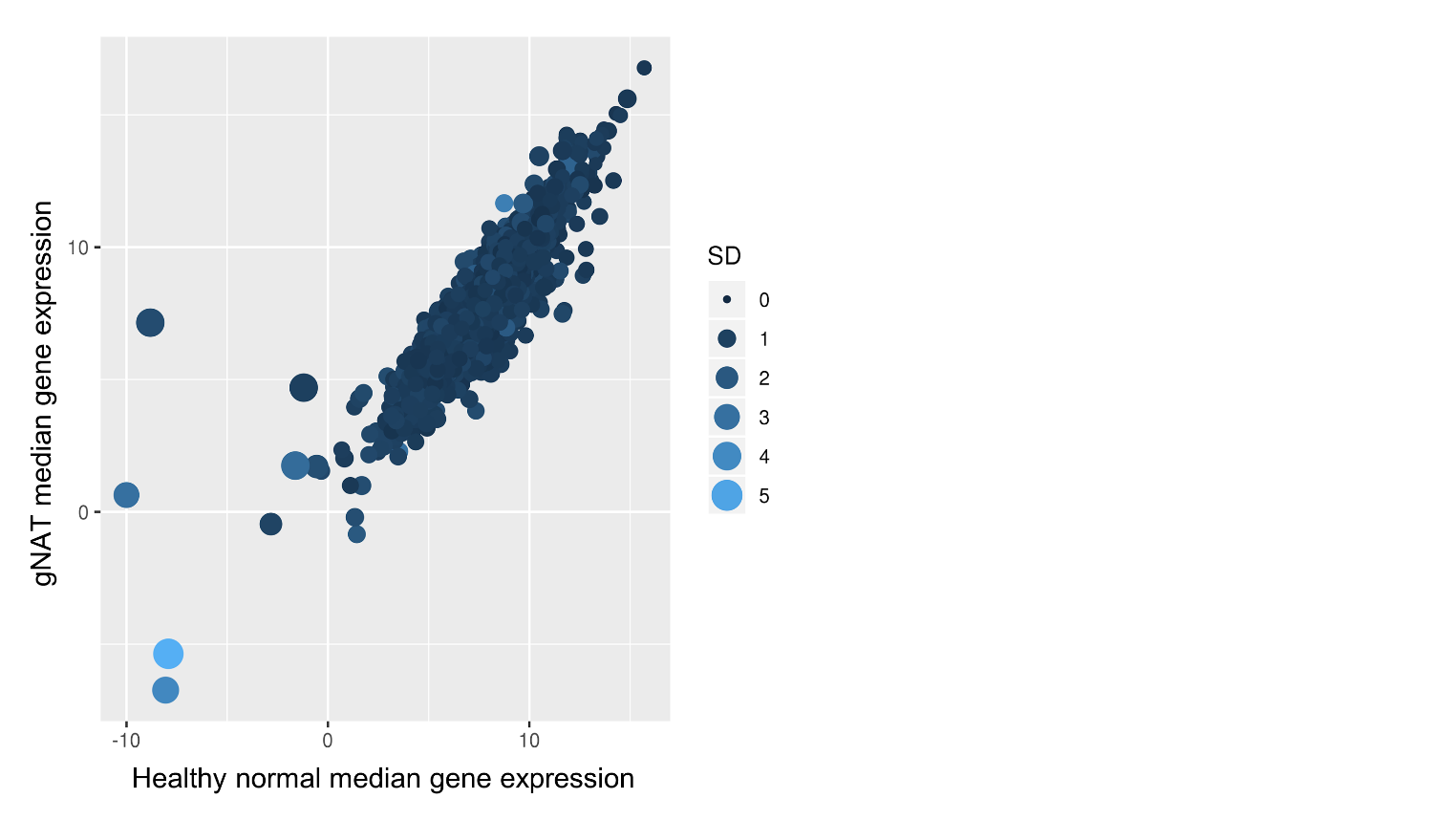


**Figure S1.** Scatter plot of the median expression levels of housekeeping genes in normal and gNAT samples. Greater the standard deviation (SD), greater the radius of the points


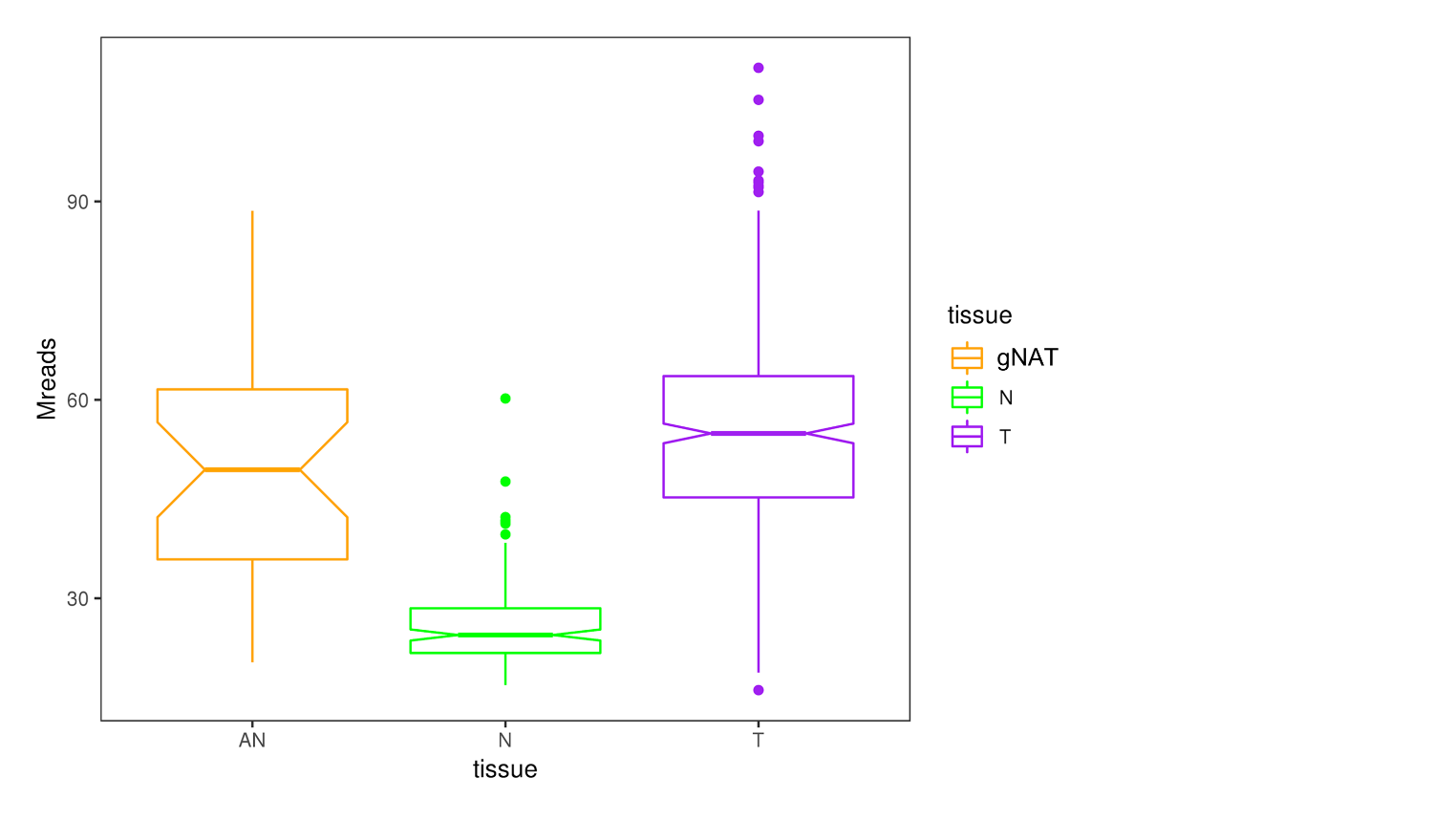


Figure S2: Boxplot of the library reads in TCGA GC and gNAT and in GTEx normal samples; GC in purple, gNAT in orange and normal tissue in green


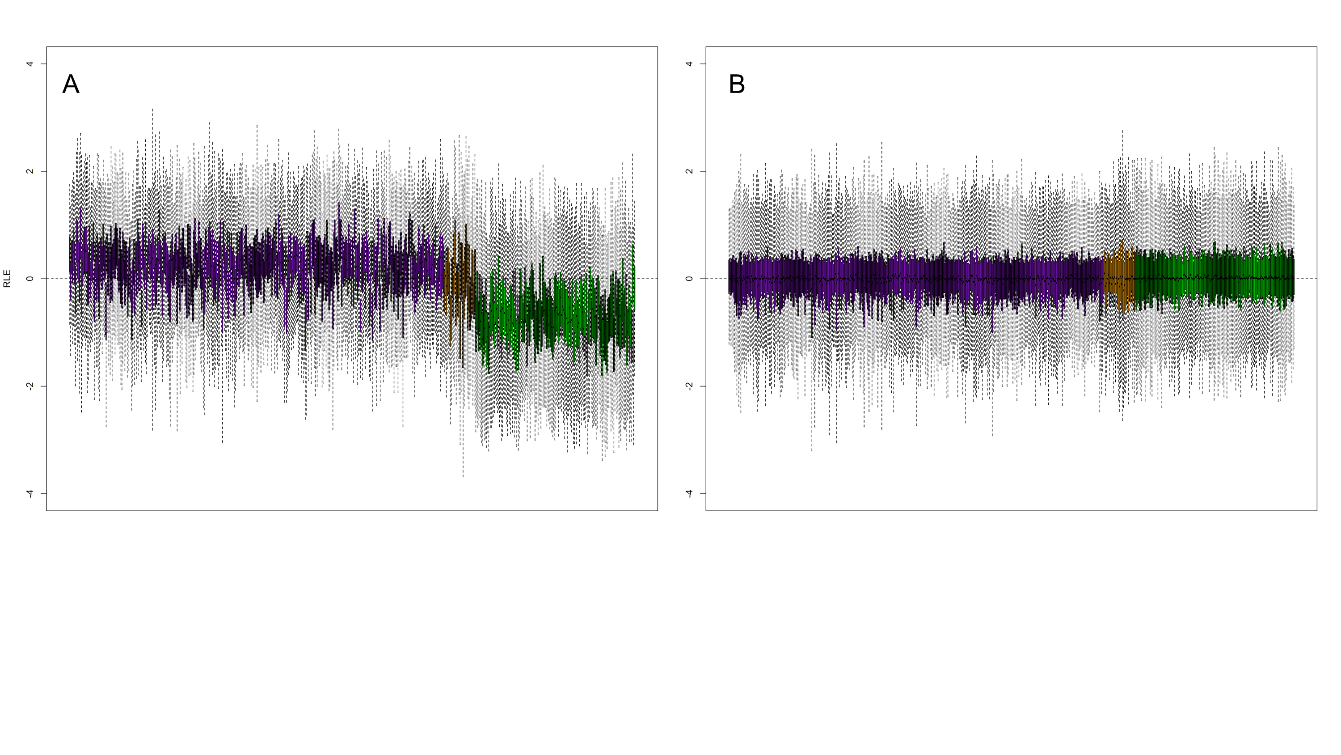


**Figure S3**. Boxplot of the relative log expression (RLE) pre and post (EDAseq) normalization. A shows the relative log expression (RLE) without normalization. There are differences in RLE between TCGA (GC (purple) and gNAT (orange)) samples and GTEx healthy samples (green) while in B, after normalization, no apparent differences observed


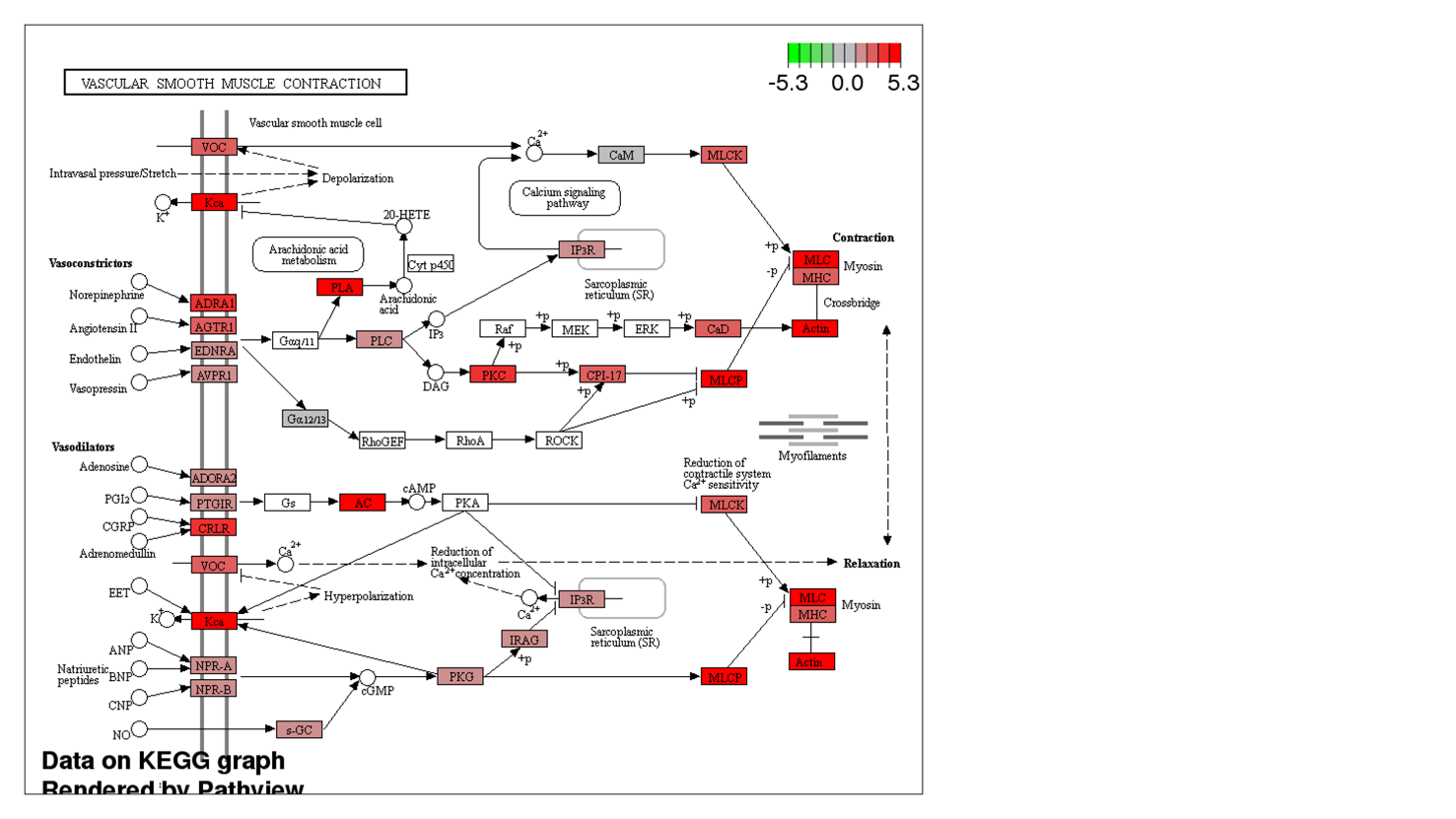


**Figure S4**. KEGG enrichment of the vascular smooth muscle contraction pathway (hsa04270) using genes up-regulated in normal muscular vs normal mucosa


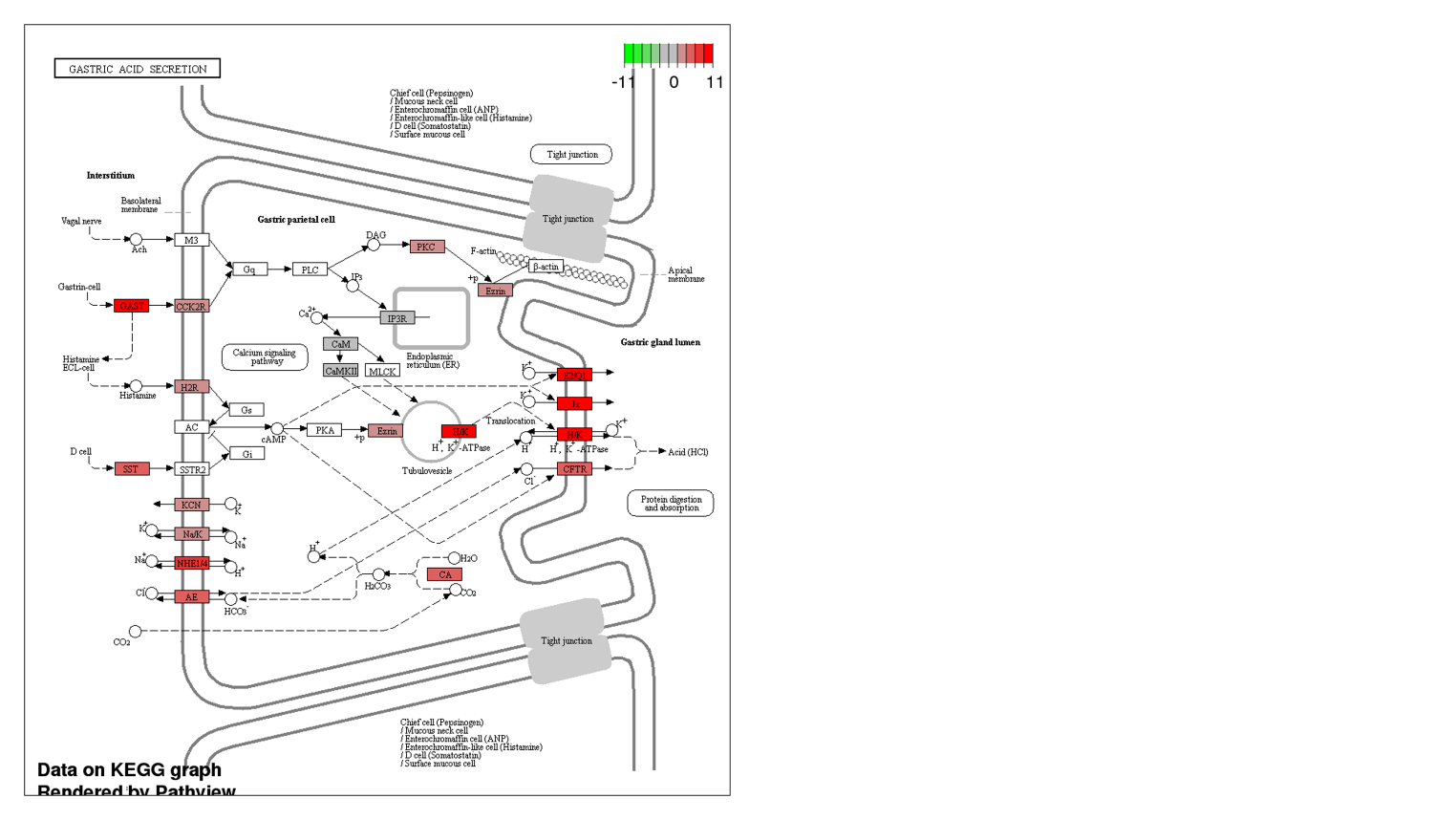


**Figure S5** KEGG enrichment of the gastric acid secretion pathway (hsa04971) using genes up-regulated in normal mucosa vs normal muscular
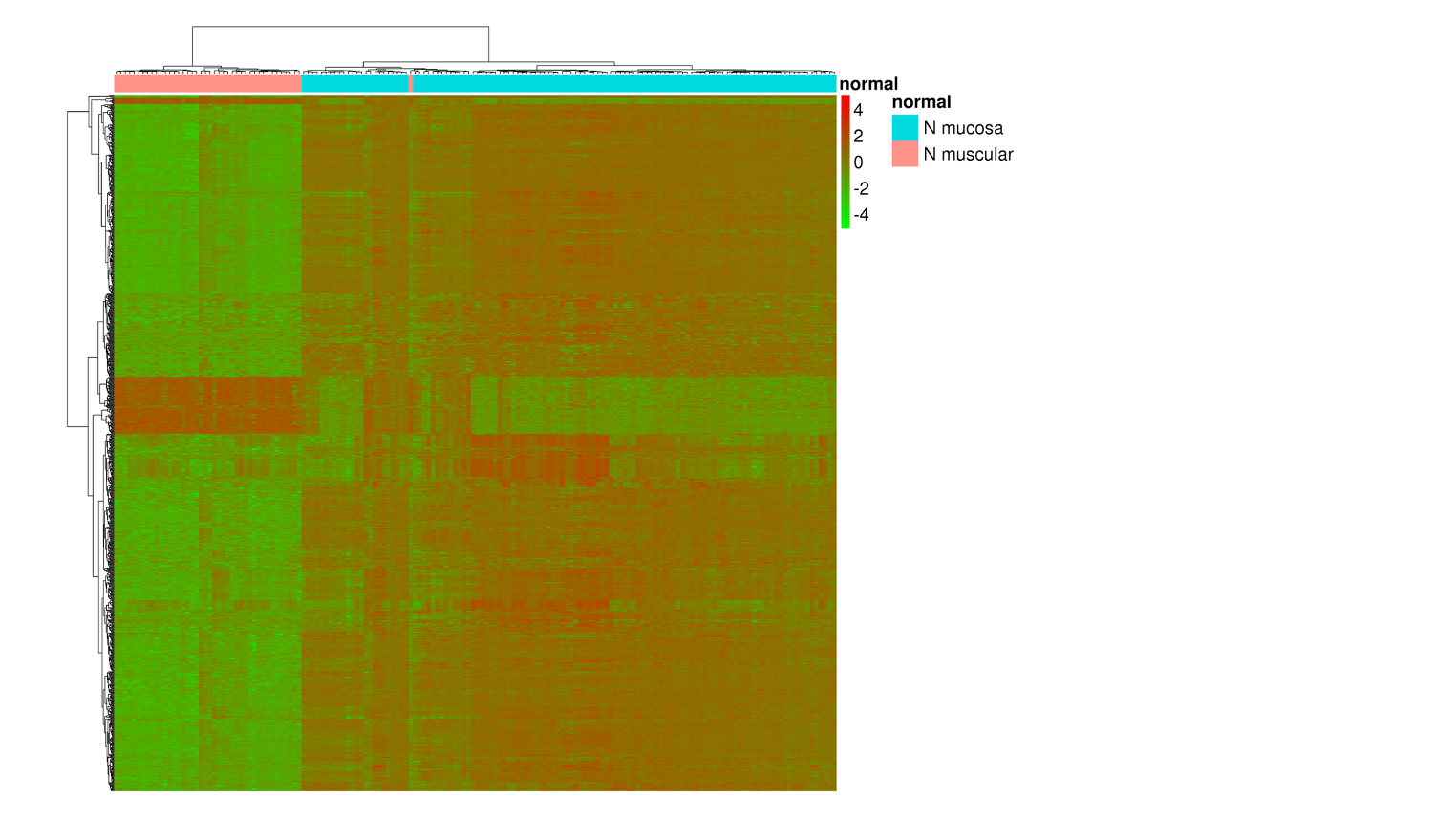


**Figure S6.** Heat map of the top 900 DEGs between normal mucosa vs normal muscular samples. log2FCs in red-green color scale. In legend, above the HM, normal mucosa in cyan while normal muscular in red


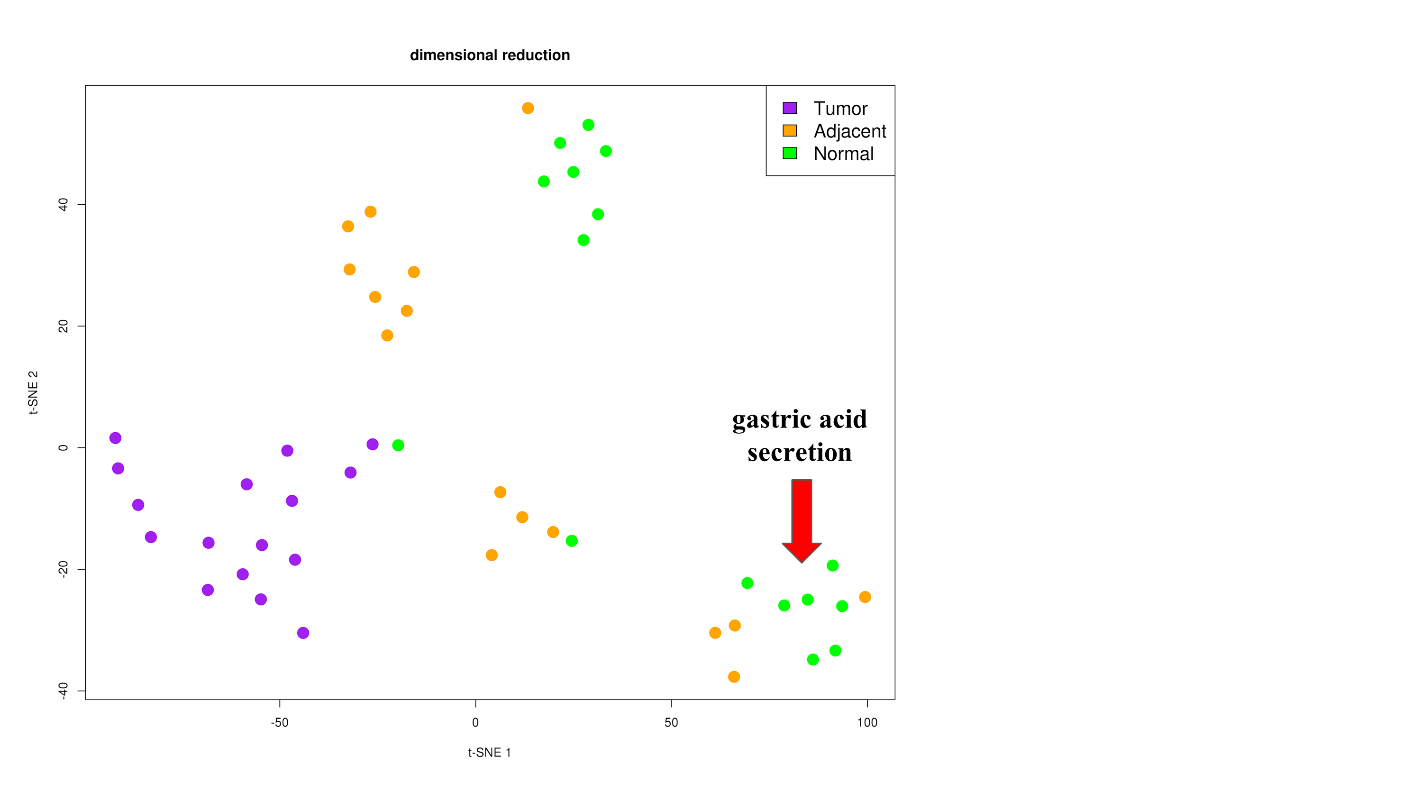


**Figure S7.** Scatter plot of the E_MTAB_1338 paired samples after dimensionality reduction procedure. A) GC in purple, gNAT in orange and normal tissue in green


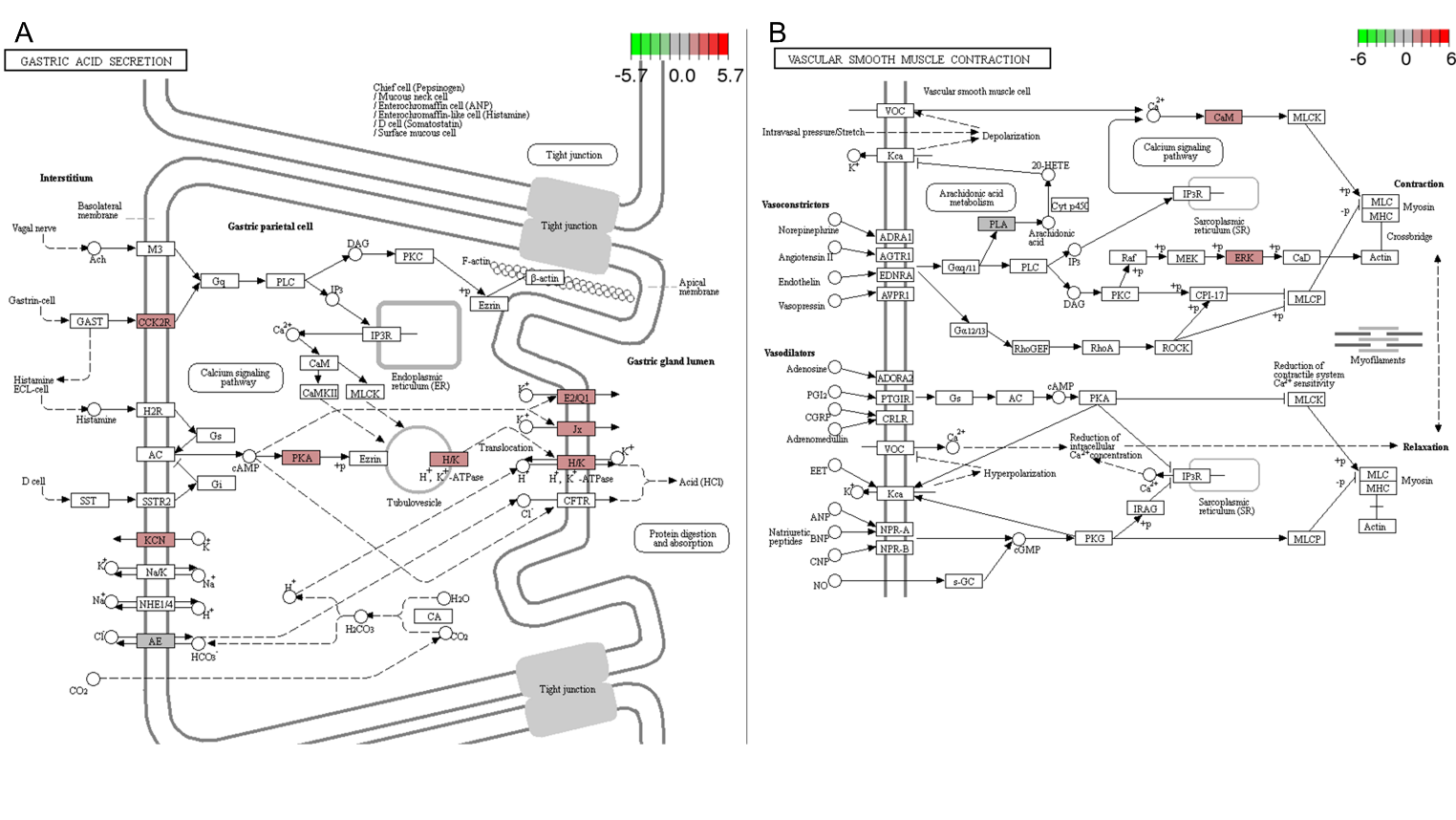


**Figure S8.** KEGG pathways enrichment in E_MTAB_1338 database using DEGs between the right-low cluster and the middle-high cluster of normal samples, respectively. A) Gastric acid secretion pathway (hsa04971). B) Vascular smooth muscle contraction pathway (hsa04270). Only the Gastric acid secretion pathway is significantly enriched.


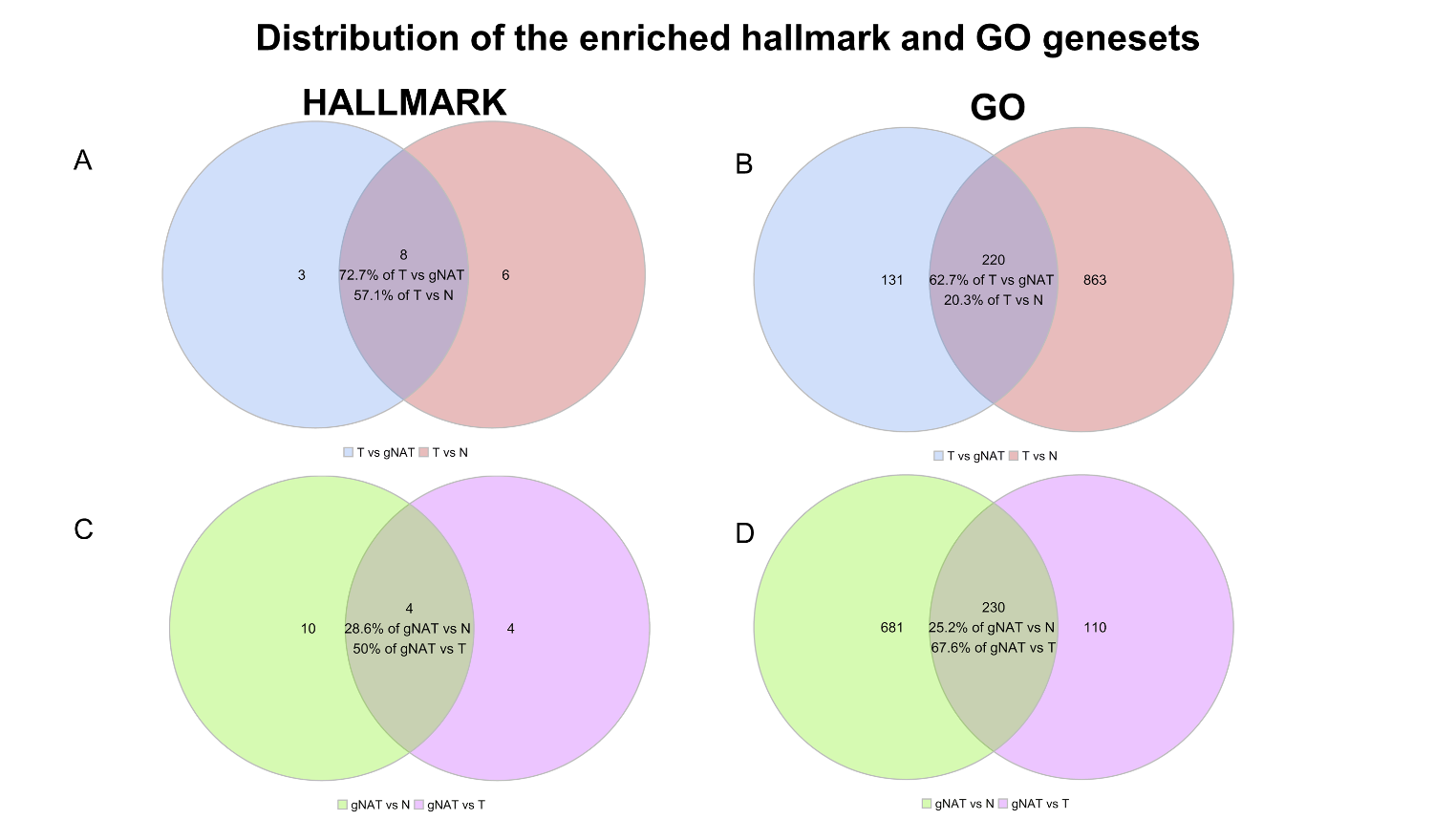


**Figure S9.** Overlap of the enriched hallmark and GO gene sets; A and B depict the results of the overlap between gNAT vs normal and gNAT vs tumor; C and D depict the results of the overlap between tumor vs gNAT vs tumor vs normal.


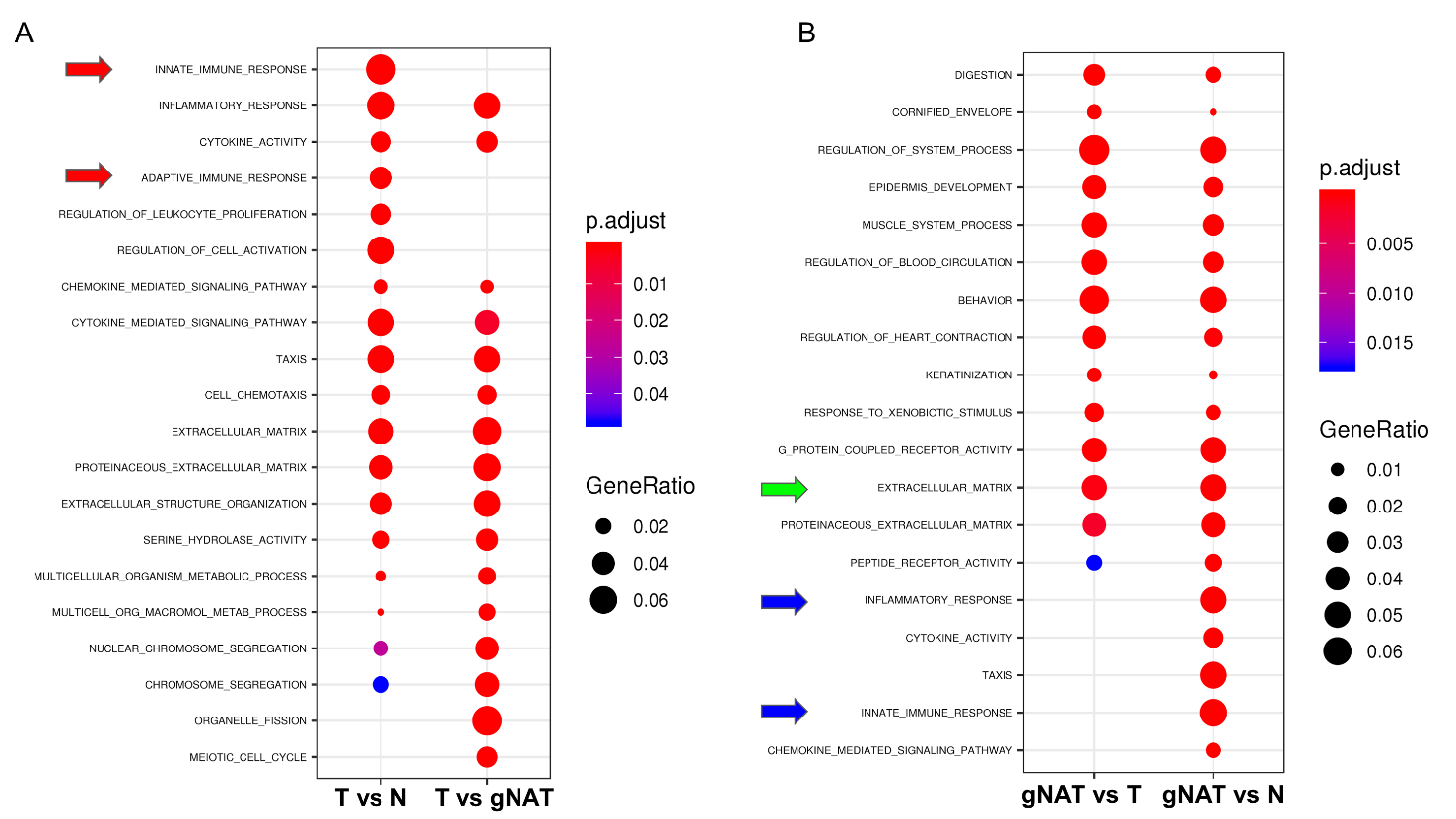


**Figure S10.** gene set analysis (GSA) of the GO categories using the DEGs between tumor or gNAT vs each of the other 2 tissues, respectively. Adjusted p-value in red-blue color scale. Gene ratio in dot size scale. Red, blue and green arrows highlight interesting exclusive and common gene sets, respectively. A) Tumor centered analysis, B) gNAT centered analysis.


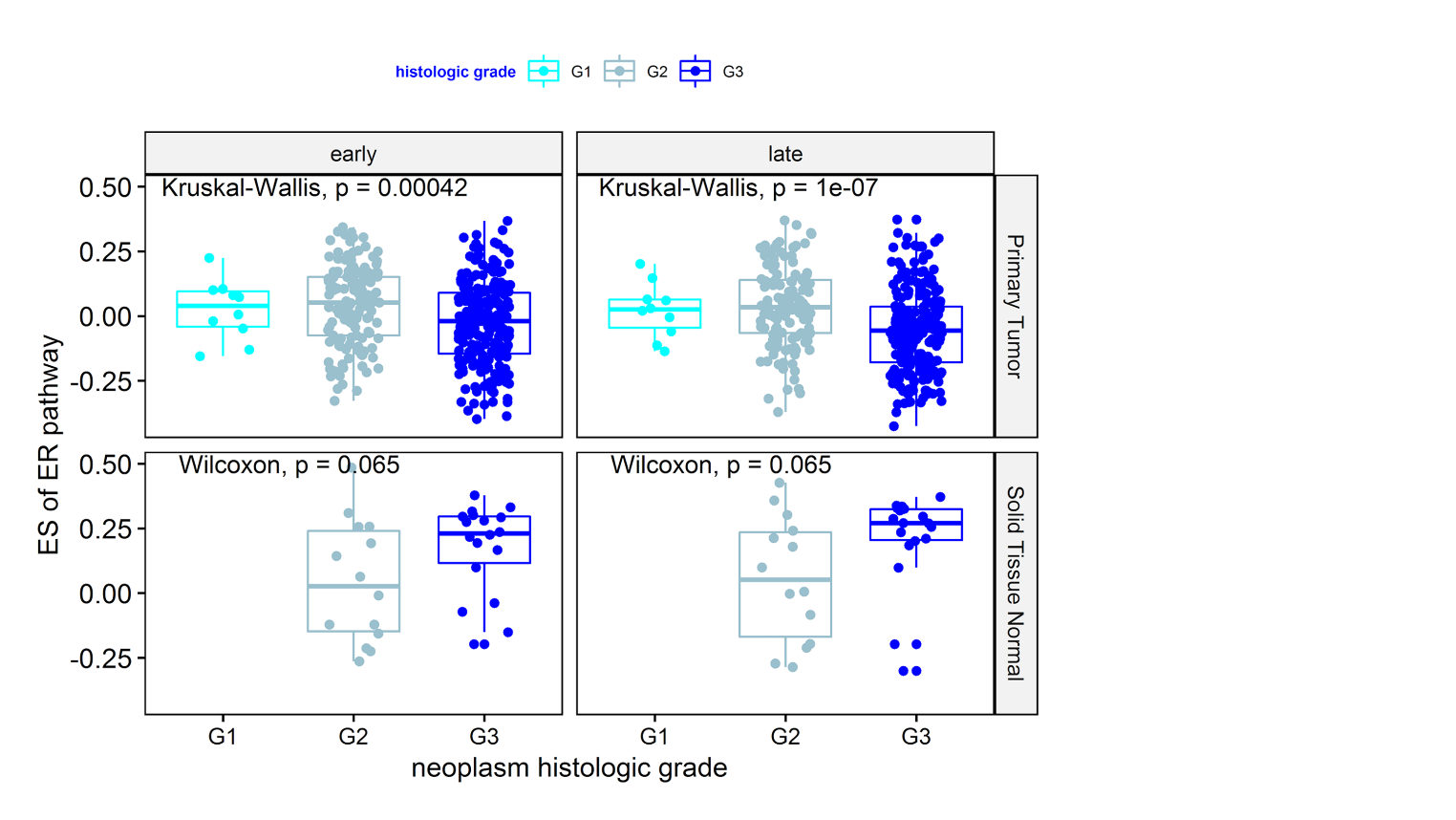


**Figure S11.** Association of the histological grade with hallmark early and late ER pathways activity in gNAT and tumor
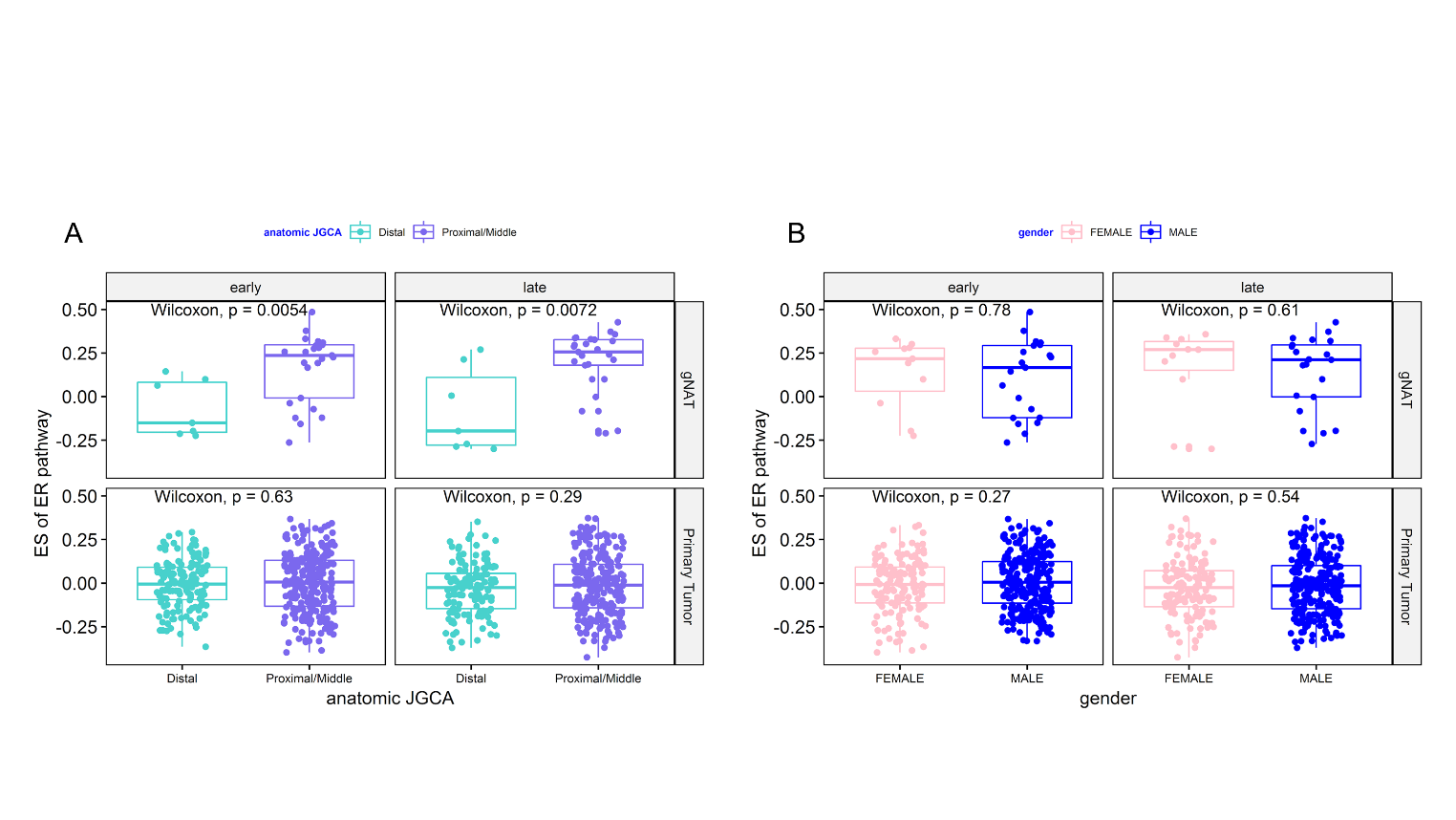
 **Figure S12.** Association of JGCA anatomical part (A) and gender (B) with hallmark early and late ER pathways activity in gNAT and GC


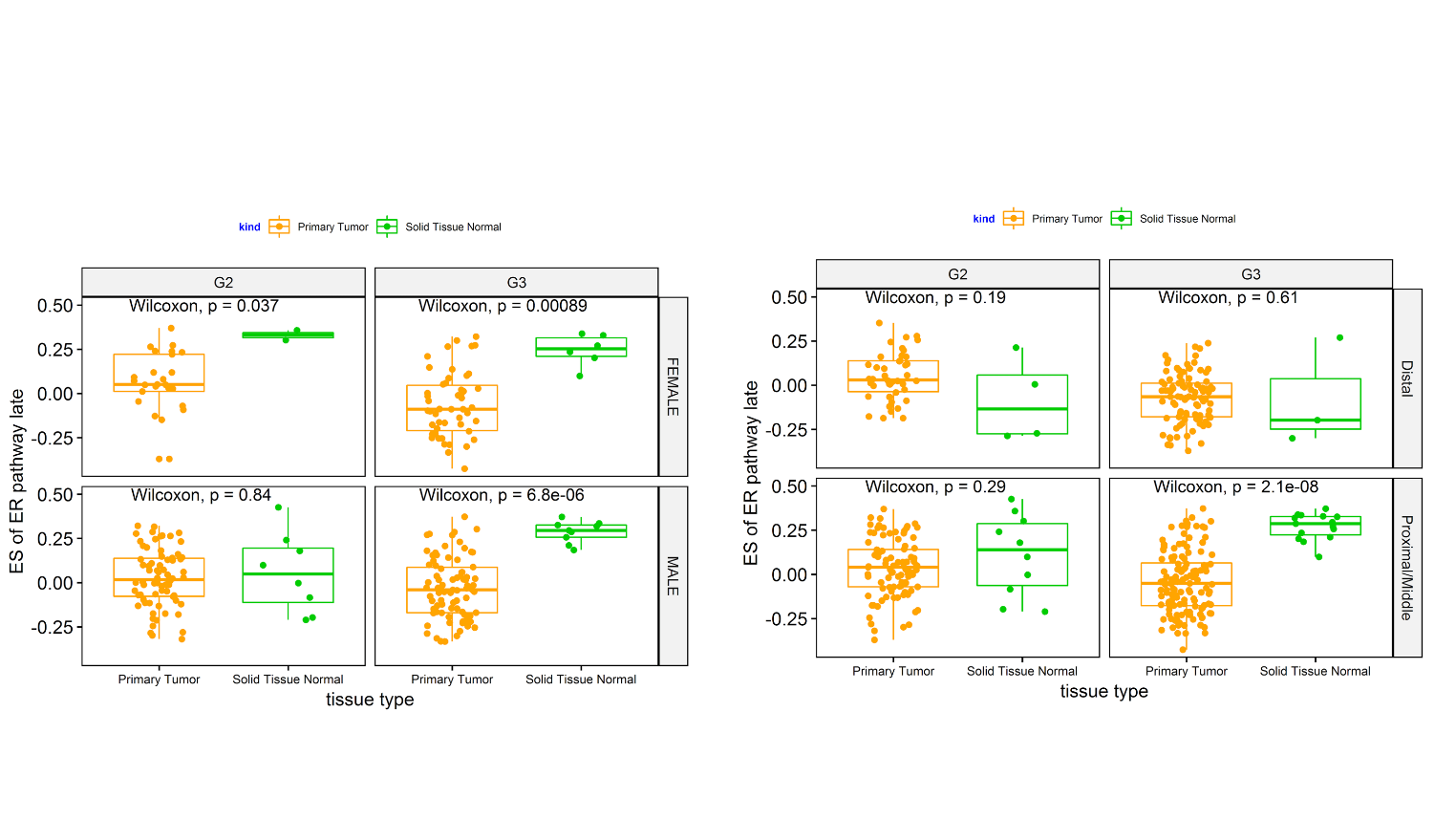
 **Figure S13.** Association of therapy tissue type and grade with hallmark late ER pathway activity according to gender (A) or JCGA anatomical site (B), respectively


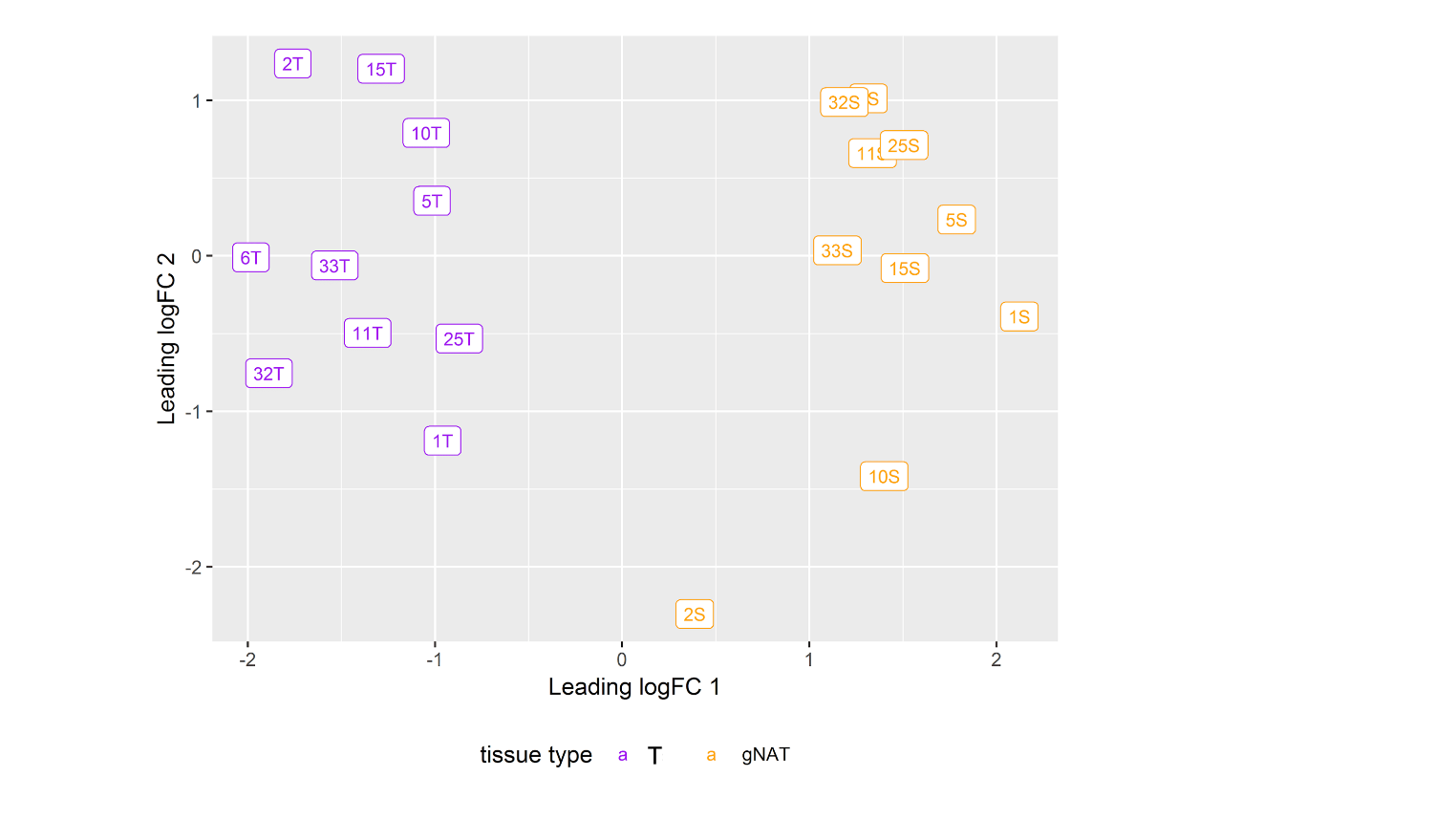


**Figure S14.** Multidimensional scaling plot of the RNA samples in which distances correspond to leading log-fold-changes between each pair of RNA samples. Tumor in purple, gNAT in orange, labels represents the name of the samples.

**Table S1**. Summary of the differentially expressed genes among adjacent, normal and tumor samples.

|  | **TOT** | **up-regulated** | **down-regulated** |
| --- | --- | --- | --- |
| **Adjacent vs Normal** | 5415 | 3711 | 170*4* |
| ***Adjacent vs Tum*or** | **33**30 | *1*797 | 1533 |
| **Tumor vs Normal** | 5248 | 3646 | 1602 |

**Data S1.** TCGA DEGs tumor vs adjacent vs normal

**Data S2.** Enriched hallmark in TCGA gNAT

**Data S3.** Univariate analysis of the most interesting clinical variables (grade, TNM, anatomic positions, etc.) on the TCGA data

**Data S4.** inHouse DEGs in GC vs gNAT
